# NetMHCpan-3.0; improved prediction of binding to MHC class I molecules integrating information from multiple receptor and peptide length datasets

**DOI:** 10.1186/s13073-016-0288-x

**Published:** 2016-03-30

**Authors:** Morten Nielsen, Massimo Andreatta

**Affiliations:** Instituto de Investigaciones Biotecnológicas, Universidad Nacional de San Martín, Buenos Aires, Argentina; Center for Biological Sequence Analysis, Technical University of Denmark, Kgs. Lyngby, Denmark

## Abstract

**Background:**

Binding of peptides to MHC class I molecules (MHC-I) is essential for antigen presentation to cytotoxic T-cells.

**Results:**

Here, we demonstrate how a simple alignment step allowing insertions and deletions in a pan-specific MHC-I binding machine-learning model enables combining information across both multiple MHC molecules and peptide lengths. This pan-allele/pan-length algorithm significantly outperforms state-of-the-art methods, and captures differences in the length profile of binders to different MHC molecules leading to increased accuracy for ligand identification. Using this model, we demonstrate that percentile ranks in contrast to affinity-based thresholds are optimal for ligand identification due to uniform sampling of the MHC space.

**Conclusions:**

We have developed a neural network-based machine-learning algorithm leveraging information across multiple receptor specificities and ligand length scales, and demonstrated how this approach significantly improves the accuracy for prediction of peptide binding and identification of MHC ligands. The method is available at www.cbs.dtu.dk/services/NetMHCpan-3.0.

**Electronic supplementary material:**

The online version of this article (doi:10.1186/s13073-016-0288-x) contains supplementary material, which is available to authorized users.

## Background

Binding of peptides to MHC (major histocompatibility complex) molecules is a prerequisite for a peptide to be an immunogen. The MHC class I molecule is highly specific, engaging in binding with only a minute proportion of the peptides offered through the antigen presentation pathway [1]. This property makes binding to the MHC molecule the single most selective step in antigen presentation. Given the pivotal role of the MHC, significant efforts have been dedicated to the development of methods capable of accurately predicting this event. These methods generally fall into two groups: allele-specific, where a method is trained for every individual MHC molecule; and pan-specific, where a single method is trained on data covering multiple MHC molecules. In particular, the latter methods have proven very powerful as they allow for binding predictions to all MHC molecules, including those characterized with limited or no binding data [2, 3]. This aspect becomes extremely important if we consider the huge polymorphism of MHC genes, with several thousand allelic variants identified to date in the HLA loci [4]. Examples of the two types of prediction tools are (reflected by high performance in the IEDB weekly automated MHC class I benchmark [5]): allele-specific (*NetMHC* [6, 7], *SMM* [8, 9]) and pan-specific (*NetMHCpan* [2, 3], *NetMHCcons* [10]). Note that many other tools have been proposed, but it is out of the scope of the paper to review them all.

Most accurate methods for prediction of binding to MHC are data-driven, meaning that the methods are trained on peptide data with experimental information about the binding affinity to the MHC molecule in question. A prerequisite for the development of accurate data driven prediction method is the availability of large and accurate datasets [11]. All the methods mentioned above obtain these data from the IEDB. These data have a large bias toward peptides of length 9 (>73 % of the data are for 9mers, whereas <3 % of the data are for peptides of length 11) (http://tools.immuneepitope.org/main/datasets, dataset used for retraining the IEDB class I binding prediction tools). This bias in peptide length has great implications for the accuracy of binding predictions as the performance of the methods in general will be poor for lengths different from nine. We have previously suggested a simple approximation approach that uses neural networks trained on 9mer data to extrapolate predictions for peptides of lengths other than 9 [12]. This approximation is currently used in the *NetMHCpan* method. However, in the context of *NetMHCpan* this approach is clearly suboptimal as it completely ignores the information contained in data with a peptide length different from nine amino acids.

Many MHC binding prediction methods suffer from another serious limitation: they cannot capture the preferences of MHC allelic variants in terms of peptide length (what we will call the “length profile”). Even though most MHC molecules prefer to bind 9mer peptides, experimental data have demonstrated that the length profiles differ substantially between MHC molecules, with prominent examples being the mouse H-2-Kb with a preference for eight amino acids-long peptides [13] and HLA-A*01:01 where close to 35 % of bound peptides have a length longer than nine amino acids [14]. For an allele-specific method, we have recently demonstrated how a simple alignment step allowing for insertions/deletions in the peptide data could integrate information across peptides of different lengths into one prediction method, not only leading to improved prediction accuracy in terms of binding affinity but also capturing differences between MHC molecules in terms of preferred length of the bound peptides [15].

Here, we incorporate this new alignment step in the training strategy with the goal to investigate whether it can boost the predictive performance also in the pan-specific case by integrating information from datasets comprising peptides of different lengths. We further analyze if the pan-specific approach can capture the differences in the length profile of different MHC molecules, and quantify to what degree the method leads to improvement in performance when predicting MHC ligands. Lastly, we apply the new method to address the issue of the binding threshold for optimal identification of MHC ligands, and investigate whether a percentile rank threshold rather than an affinity threshold is optimal when performing rational epitope screening across multiple MHC molecules.

## Implementation

### Data

The MHC class I binding dataset was downloaded from the IEDB [16] (http://tools.immuneepitope.org/main/datasets; dataset used for retraining the IEDB class I binding prediction tools). This dataset consists of 186,684 peptide-MHC binding affinity measurements covering 172 MHC molecules from human, mouse, primates, cattle, and swine. We introduced 25 random natural peptides for each of the lengths 8, 9, 10, and 11 as artificial negatives for each allele, to ensure a sufficiently diverse set of negative examples [3]. These random sequences were only used for training and were excluded from all evaluations. The data were split into five partitions for cross-validation as described earlier [17] to ensure that no identical 8mer segment was shared between partitions.

### Network training and architecture

Networks were trained as previously described [3], encoding each MHC molecule in terms of a pseudo sequence in order to leverage information between MHC molecules. Moreover, we extended this pan-specific approach by allowing insertions and deletions in the multiple sequence alignment as described by Andreatta and Nielsen [15]. In short, the amino acid sequence of training examples was Blosum encoded using 20 values corresponding to the BLOSUM matrix scores vector [7]. Peptides longer than nine amino acids were reduced to a core of nine amino acids by applying consecutive amino acid deletions. These included both deletions at the end terminals and consecutive deletions within the peptide. In the case of peptides shorter than nine amino acids, a wildcard amino acid X (encoded as a vector of zeros) was inserted to extend the peptide to a 9mer core. Deletions and insertions were attempted at all possible locations within the peptide and the configuration returning the highest predicted score was saved as the optimal binding core. The current best solution was used together with the MHC pseudo sequence for error back-propagation and the procedure was iterated.

Other features of the training examples that were presented to the neural networks are: the length of the deletion/insertion; the length of peptide flanking regions, which are larger than zero in the case of a predicted extension of the peptide outside either terminus of the binding groove; and the length L of the peptide, encoded with four input neurons corresponding to the four cases L < =8, L = 9, L = 10, L > =11. As for the original *NetMHCpan* method, the hidden layer of the networks consisted of 56 or 66 hidden neurons and the output layer of one neuron having as target value the binding affinity of the training example rescaled between 0 and 1 using the relationship 1-log(aff)/log(50,000), where aff is the IC50 affinity value in nM units [7].

Networks were trained in five-fold cross-validation using gradient descent back-propagation with early stopping. Ensembles were generated by training five networks for each data partition and network architecture each starting from a distinct random initial configuration, leading to an ensemble of 10 networks for each data partition, and a total of 50 networks across all partitions. The ensemble trained on all alleles and all peptide lengths will be referred to as the “allmer” method.

For comparison, a network ensemble was trained using only the subset of 9mer peptides (“9mer” method) from the five data partitions described above. As in NetMHCpan-2.8, the L-mer approximation described by [12] was used for the networks trained on 9mer data only to extrapolate predictions for peptides of length different from nine. The L-mer approximation relies on networks trained only on 9mers, inserting/deleting amino acids at non-anchor positions in shorter/longer query peptides to conform the peptides to a series of 9mers and then averaging the predictions of the 9mer sequences.

Likewise, an ensemble of allele-specific networks was trained on peptides of multiple lengths (“allmer-allele” method) using the *NetMHC-4.0* method described recently by Andreatta and Nielsen [15].

### SYFPEITHI evaluation data

As an independent evaluation set, we extracted a set of 2329 unique MHC class I ligands of length 8–11 from the SYFPEITHI database [14], excluding all peptide –MHC pairs found in the training set. To remove potential noise imposed by wrong annotation of the MHC restriction element and/or incorrect definition of the minimal ligand binding core, a filter was applied as previously described [15] and all peptide-MHC pairs with a predicted rank score >10 % (calculated using both *NetMHCpan-2.8* and the pan-specific allmer method developed here) were removed, resulting in a set of 2147 MHC ligands. The source protein sequence of each validated ligand was scanned with a sliding window of 8–11 amino acids to generate all possible 8, 9, 10, and 11mers contained in the protein. These overlapping peptides were then ranked by predicted binding affinity, and for each protein we measured the relative rank of the validated ligand in the list of affinity predictions. The rank of the known ligand measures the fraction of peptides in the protein that would have to be tested before identifying the actual positive and can be used as a metric of predictive performance.

### Statistical tests

The predictive performances of alternative methods are compared using binomial tests. The null hypothesis is that either of the two methods being compared has equal probability of returning higher PCC (or AUC) on a given MHC allele. If method 1 has higher PCC in n1 alleles and method 2 higher PCC in n2 alleles, we estimate the *p* value of this event as the probability of observing n1 or more wins by chance in a binomial distribution B(n1 + n2, 0.5). Note that ties are excluded.

### Information divergence

The information divergence is calculated as $I={\sum}_a\kern0.5em {f}_a\kern0.5em  \log \left(\frac{f_a}{b_a}\right)$, where the sum is over the alleles included in the analysis, *f*_*a*_ is the observed frequency of allele *a* (the proportion of peptides predicted to bind allele *a*), and *b*_*a*_ is the background frequency of allele *a* (the proportion of peptides with predicted binding to allele *a* irrespectively of binding value).

## Results

We have previously demonstrated how a pan-specific training approach that allows for leveraging of binding information across multiple MHC molecules leads to a significant boost in predictive performance for alleles covered with limited or even no binding data [2]. Likewise, we have recently shown that a method exploiting binding information from peptides of different lengths can boost the predictive performance for all peptide lengths, in particular for those covered by limited binding data [15]. In this work, we aim to investigate whether combining these two approaches into a pan-allele, pan-length training pipeline would lead to a further improvement in predictive performance for MHC class I binding prediction.

### Comparing a pan-specific method trained on 9mer data only (9mer) to a pan-specific method trained on data covering multiple lengths (allmer)

**Fig. 1:**
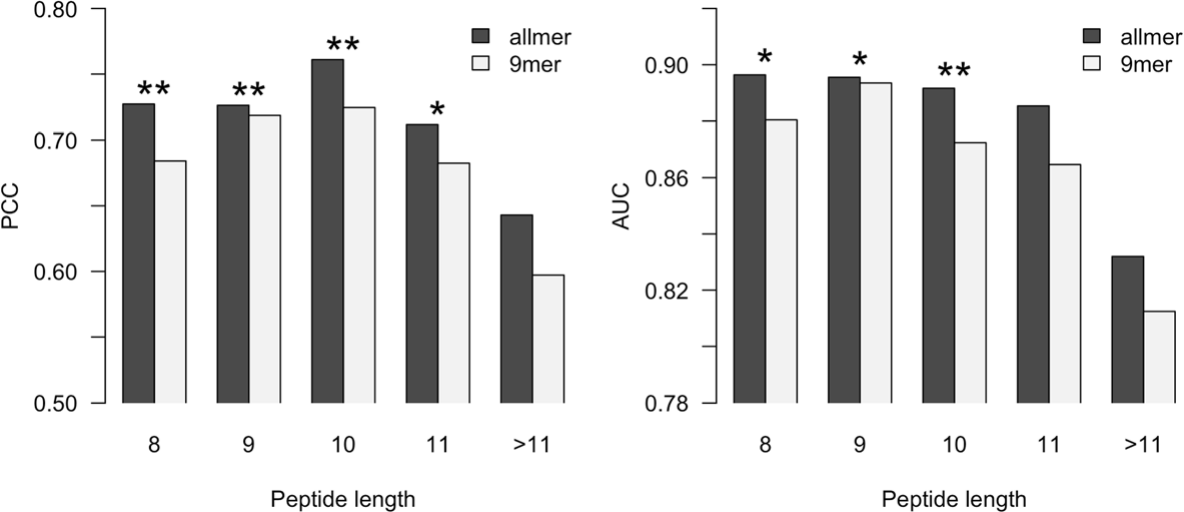
Predictive performance on different peptide lengths for the allmer and 9mer predictive methods. The two methods were trained as described in the text. The predictive performance was measured in terms of Pearson’s correlation coefficient (PCC) and area under the ROC curve (AUC), the latter using a binding threshold of 500 nM. The allmer method significantly outperforms the 9mer approach on peptides of all lengths from 8 to 10 (binomial test excluding ties). **: p < 0.001, *: p < 0.05

Two ensembles of pan-allele networks were trained and evaluated using cross-validation as described in Materials and Methods: allmer includes all binding data from the IEDB dataset and 9mer includes only 9mer data. For each allele-length combination characterized with at least three binders (defined using a threshold of 500 nM) and 20 data points, the predictive performance was estimated in terms of the Pearson’s correlation coefficient (PCC) and area under the ROC curve (AUC). Figure 1 shows the average performance values for the two methods for different peptide lengths (results for all alleles are available in Additional file 1).

The allmer method outperformed the 9mer method at all length scales. The difference is statistically significant at all length scales except for peptides of length 11 or longer (binomial tests excluding ties).

### Comparing an allele-specific method trained on peptides of multiple lengths (allmer-allele) to a pan-specific method trained on peptides of multiple lengths (allmer)

**Fig. 2:**
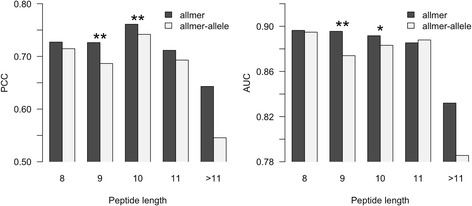
Predictive performance on different peptide lengths for the allmer and allmer-allele predictive methods. The two methods were trained as described in the text. The predictive performance was measured in terms of Pearson’s correlation coefficient (PCC) and area under the ROC curve (AUC), the latter using a binding threshold of 500 nM. The allmer method significantly outperforms the allmer-allele approach for peptides of length 9 and 10 (binomial test excluding ties). **: *p* <0.001, *: *p* <0.05

Next, we compared the predictive performance of the allmer networks (pan-specific when it comes to both alleles and peptide length) to a method (allmer-allele) that is trained in an allele-specific manner on data covering multiple peptide lengths (i.e. pan-specific only when it comes to the peptide length). Here, the allmer networks were trained as described above, and the allmer-allele method as described for *NetMHC-4.0* [15]. As above, the predictive performance was estimated in terms of the PCC and AUC for each allele-length combination characterized with at least three binders (defined using a threshold of 500 nM) and at least 20 data points shared between the two datasets. Figure 2 shows the average performance values for the two methods for different peptide lengths (results for all alleles are available in Additional file 2).

The allmer approach showed higher average PCC and AUC on most peptide lengths, although the difference is statistically significant only for 9mers and 10mers. Focusing in particular on the subset of alleles characterized by few data points (less than five peptide binders), we find that the allmer method consistently (16 out of 18 cases) achieves a higher predictive performance in terms of PCC compared to the allmer-allele method (data in Additional file 2). This result confirms the earlier finding that the pan-specific training procedure is capable of leveraging information across different allele datasets boosting performance for alleles characterized by limited experimental data [3].

### Peptide length preferences of MHC binders

**Fig. 3:**
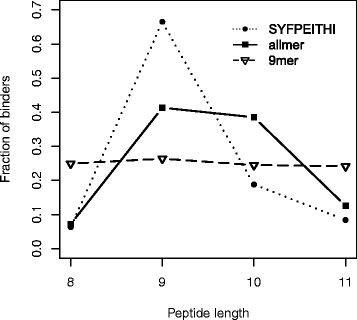
Length preference for the allmer and 9mer prediction methods compared to the length preference in the SYFPEITHI data. Length profiles for the allmer and 9mer methods were estimated as described in the text. The SYFPEITHI length preference was estimated as the average over the allele-specific length preference of 24 MHC molecules characterized by 20 or more ligand data points

Having demonstrated the superior performance of the allmer method compared to the allele-specific and length-specific versions, we proceed to investigate the peptide length preferences of individual MHC molecules. On the set of 24 MHC alleles characterized with 20 or more ligand data in the SYFPEITHI database, we predicted binding affinity values for 1,000,000 random natural peptides with a length of 8–11 amino acids (250,000 peptides for each length) using the allmer and 9mer models (using the L-mer approximation to predict binding for non-9mer peptides). We estimated a peptide length histogram of the MHC molecules by taking the top 1 % (10,000) predicted binders to each MHC allele. Figure 3 shows the average length histograms for the two methods. For comparison, we included in the graph the average of the length histograms estimated from the ligand data in the SYFPEITHI database (the complete set of allele-specific histograms for the three methods is found in Additional file 3).

The results displayed in Fig. 3 show that: (1) MHC molecules in general have a pronounced preference for presenting 9mer peptides; (2) the conventional 9mer method based on the L-mer approximation does not capture this preference and predicts a close to uniform fraction of binders at all peptide lengths; and (3) only the allmer approach, leveraging information across peptide lengths, can predict a length preference that follows the length distribution trend of experimental data.

These observations are in agreement with earlier findings derived from an allele-specific training pipeline which made use of peptides of multiple lengths [15].

**Fig. 4:**
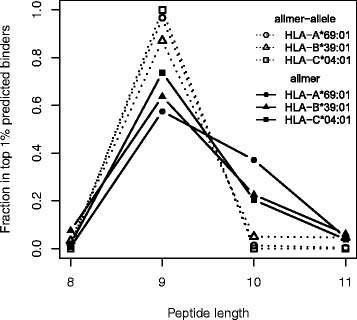
Comparison of the predicted length profile for alleles characterized by no or limited peptide data of length different from nine amino acids. The distribution of predicted binders for the three alleles were characterized by relatively large data sets (>500 data points) with more than 99 % 9mers. Length profiles were estimated from the top 1 % of 1,000,000 random natural 8–11mer peptides using the allmer-allele (the method trained on allmer data in an allele-specific manner), and the allmer (the pan-specific method trained on allemer data) methods, respectively

Focusing once more on alleles characterized by limited data and in particular datasets with limited data available for non-9mer peptides, we find that, in contrast to a method trained in an allele-specific manner, the allmer approach also for these alleles predicts a length profile tolerating non-9mer binders (see examples of such alleles in Fig. 4). The length profiles obtained from elution data from the SYFPEITHI and IEDB databases for the molecules HLA-B*39:01 and HLA-C*04:01 (only three ligand data points are available for HLA-A*69:01) are close to identical with a preference for 9mers (74 %) followed by 8mers (13 %) and 10mers (10 %). Although the predicted distribution does not coincide perfectly with the distribution of eluted peptides, the allmer method clearly provides a better description of the length profile compared to the allele-specific method. The pan-specific method has therefore the powerful property of inferring a length profile of a given MHC molecule even with scarce experimental data. In the next section, we investigate how this aspect can positively affect the identification of new MHC ligands.

### Identifying MHC ligands

**Fig. 5:**
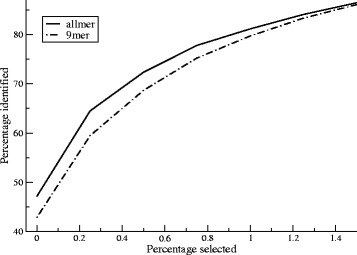
Rank analysis on the SYFPEITHI ligand benchmark. Binding to the restriction element was predicted for all 8–11mer peptides within the source proteins from the SYFPEITHI data set using the allmer and 9mer prediction methods, respectively. The percentage of identified ligands is plotted as a function of the percentage of top predicted binders from each source protein-ligand-MHC combination

Next, we aimed to quantify how the gain in predictive performance brought by the pan-allele/pan-length approach translates into reduction of cost for identifying MHC ligands. We obtained a dataset of 2154 ligands from the SYFPEITHI database consisting of 8–11mer ligands with known MHC restriction (see Materials and Methods). Using the 9mer and allmer prediction methods, we predicted binding to the given MHC restriction element for all 8–11mer peptides in the source proteins of the ligand. Next, for all MHC-ligand-source protein combinations, we extracted a given proportion of top scoring peptides (percentage selected), and calculated the percentage of the 2154 ligands contained within this selected peptide set (percentage identified). Varying the percentage selected, we can thus construct the curves presented in Fig. 5.

The analysis confirms the enhanced predictive performance of the allmer method compared to 9mer approach. If the top 0.025 % (the first point on the two curves) of predicted binders are selected for each MHC-ligand source protein, allmer identifies 47 % of the known ligands, whereas only 42 % are identified using 9mer. Similarly, if 75 % of the ligands are to be identified, 0.62 % of all 8–11mer peptides need to be tested by using allmer. This number is increased by 23 % to 0.76 % using 9mer. Although this difference is small, it translates into a substantial cost reduction in screening for peptide ligands in large datasets consisting of thousands of proteins. By way of example, the top 0.76 % of all 8–11mer peptides in a set of 1000 proteins of average length 300 amino acids would correspond to 2280 peptides. In this situation, the reduction by 23 % achieved by the allmer method will correspond to 420 peptides.

### Percentile rank score for known ligands/epitopes. How many MHC ligands are captured at different rank scores?

**Fig. 6:**
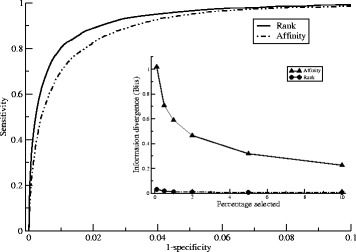
ROC curve analyses for the SYFPEITHI benchmark dataset. Binding to the restriction element was predicted for all unique 8–11mer peptides within the source proteins from the SYFPEITHI benchmark using the allmer method. Binding values were reported as binding affinity and percentile rank values as described in the text. ROC curves were calculated for each prediction value taking ligands as positives and all other peptides as negatives. The inset plot shows the information divergence value (ID) as a function of the percentage of peptides selected. The ID was calculated from the proportion of peptides with predicted restriction to each of the MHC molecules in the benchmark compared to the proportion expected by sampling at random

Earlier studies have demonstrated that different MHC molecules present ligands/epitopes at distinct binding thresholds [18, 19]. Considering the predicted binding values of the allmer method described here, this situation also holds true. We observed a large difference in the proportion of predicted peptide binders at any fixed affinity value across different MHC molecules. Limiting the comparison to HLA molecules characterized with at least 20 binders (defined using a threshold of 500 nM) and 100 data points, we find for instance that the molecule HLA-A*02:11 binds more than 7 % of random natural 9mer peptides with a binding affinity of 500 nM or stronger. In strong contrast to this, the HLA-C*04:01 molecule was predicted to bind less than 0.01 % of the same set of natural 9mer peptides at this binding threshold. Given these very large differences in binding affinity values between MHC molecules, we and others have earlier suggested that using percentile thresholds rather than binding affinity values would result in peptide selection that is less influenced by this variation in presentation threshold, and subsequently when sampling multiple MHC molecules in one peptide selection would lead to a dataset with a higher sensitivity and specificity compared to a selection based on affinity [20]. Using the SYFPEITHI dataset, we examined this issue using the allmer prediction method. The analysis was done in a similar manner to what we described above, but in addition we translated the predicted binding values to a percentile score by comparing them to the predicted binding affinities of a set of 400,000 random natural 8–11mer peptides (100,000 of each length). We performed this transformation to percentile rank scores for each allele. Next, we pooled peptides from all source proteins and MHC restrictions and from this dataset calculated ROC curves based on either affinity or percentile rank scores (Fig. 6). From this analysis, it is clear that using the percentile rank scores consistently achieves higher sensitivity at all specificity levels. In particular, we find that 91 % of the ligands are recovered with a specificity of 98 % at a rank threshold of 2 %. For the affinity selection, the sensitivity at this specificity value falls to 82 %, and the corresponding binding affinity threshold value is 1425 nM.

The reason for this dramatic change in performance when using percentile rank scores as opposed to affinity is the large diversity in the range of binding values for the different MHC molecules. This difference has been observed in earlier studies and is maintained in the method developed here [18]. If selecting peptides based on binding affinity, priority will be given to MHC molecules with generally higher affinity values compared to molecules binding peptides with lower affinity, leading to a highly unbalanced coverage of the different molecules analyzed. We extracted subsets of peptides from the total list of more than 4,800,000 peptides included in the SYFPEITHI benchmark dataset based on predicted binding affinity or percentile rank. Next, we compared the proportion of peptide-restrictions to each MHC molecule to the background distribution in the complete dataset at different affinity or rank thresholds and quantified the difference between the distributions in terms of Information divergence (ID) [21] (see Implementation). If the MHC restriction sampled in the selected subset is similar to the background distribution, the resulting ID value will be close to zero, and in case the distribution diverges from the background towards particular subsets of MHC alleles, a large positive ID value is obtained. The results of this analysis are shown in the inset to Fig. 6 and clearly demonstrate that the percentile rank selection in contrast to the affinity-based selection for all peptide subsets provides a sampling of the MHC space that is close to identical to the background MHC distribution. Based on these observations, we strongly recommend selecting candidate epitopes using percentile rank score as opposed to predicted affinity, as they ensure a selection of peptides that better represents the distribution of known peptide-MHCs.

## Discussion and conclusions

We have previously demonstrated how a simple neural network-based machine learning algorithm, NNAlign, can be effectively applied to identify binding motifs in quantitative receptor-ligands datasets [22, 23], and have with great success applied this method to learn the binding motifs and develop accurate prediction models for the MHC class II system [24–26]. Likewise, we have recently shown that the method can be extended to allow for integration of information from peptides of variable length and demonstrated how this leads to improved prediction accuracy for allele-specific models in the context of the MHC class I system, in particular for peptide lengths different from the canonical binding core length of 9 amino acids [15].

In this work, we have described how this approach could be applied to a pan-specific MHC class I binding prediction algorithm to obtain a significant improvement in predictive performance in terms of predicted peptide-MHC binders. We observed higher performance compared to both the traditional pan-specific MHC class I binding prediction method trained on 9mer peptide data only (*NetMHCpan-2.8*) and the methods trained in an allele-specific manner integrating information from peptides of multiple length (*NetMHC-4.0*).

Similarly, we quantified the ability of the method to identify experimentally confirmed MHC class I ligands. In order to recover the known ligands at a sensitivity level of 75 %, we estimated a close to 25 % reduction in the number of peptides that need to be tested compared to a conventional pan-specific method trained on 9mer data only.

We applied the new prediction method to substantiate why, when screening for potential binding peptides to multiple MHC molecules, we recommend the use of percentile rank scores rather than binding affinity values. From a large MHC class I ligand dataset, we confirmed the previous finding [19] that MHC class I molecules would be predicted to have peptide repertoires of extremely different sizes, if they were identified by a universal binding affinity threshold. We demonstrated that this unbalance in the affinity of the sampled peptide-space leads to a sub-optimal predictive performance when screening for binding peptides in a setting covering multiple MHC molecules. Relying on percentile rank score for the selection of potential ligands can correct this unbalance leading to improved predictive performance. In particular, we found that 91 % of the ligands would be recovered with a specificity of 98 % using a percentile rank score of 2 %. This sensitivity value drops to 82 % if the screening were based on affinity values.

While this study demonstrates that percentile rank scores return a higher sensitivity in MHC ligand identification compared to affinity scores (at a given specificity value), the rank score approach starts from the extreme assumption that the number of presented peptides is identical for all MHC molecules. Earlier studies covering a small set of alleles have suggested that this might not always be the case [18]. Given this, and the observations in our study, it seems plausible that the biologically relevant threshold for identification of MHC ligands is allele-specific and is based on a combination of percentile ranks and affinity scores. However, since we cannot quantify the size of the peptide repertoire of the many thousands known MHC class I molecules and hence cannot identify this biologically relevant binding threshold, we suggest using percentile rank score, as this measure outperforms affinity-based selections.

Another important property of the proposed method is the ability to predict the binding mode of the peptide to the receptor in terms of the binding core location, both in the case of non-canonical binders protruding at the termini [27] and for long peptides bulging out from the center of the MHC groove with canonical C and N terminal anchors [28]. While this property is also part of the recently published *NetMHC-4.0* method [15], it is to the best of our knowledge for the first time described here for a pan-specific MHC class I binding prediction method.

Even though we have demonstrated how the extended neural network-based machine learning *NNAlign* algorithm overall enables the development of accurate prediction models, these models are not any better than the quality of the data used to train them. This is especially true when it comes to the length distribution of binding peptides predicted by the model for different receptors. The predicted length distribution to a very high degree reflects the length distribution in the data used to train the model. If this distribution is at odds with the “true” distribution of the given molecule, then the predicted length distribution will also give a poor reflection of the peptide length preference of the receptor. We can hope that having developed a machine-learning algorithm that readily can handle peptide datasets of multiple lengths, the scientific community will benefit from this and expand the length space of peptides used for experimental characterization of MHC molecules to ensure that these match more closely what would be found in the biological setting. Indications of such length preferences could be obtained from various experimental resources including peptide libraries scans [29] and peptide MHC mass spectrometry elution [30, 31].

In summary, we have applied the extended neural network-based machine-learning algorithm to develop a pan-specific prediction model for the MHC class I binding system. It is clear that the application of this algorithm is not limited to this system, and that it potentially can be applied to a wide range of other receptor-ligand system characterized with quantitative data including but not limited to MHC class II, SH2, SH3, and PDZ receptors.

## Availability and requirements

The allmer method trained on the complete IEDB binding dataset is made freely available as a webserver at www.cbs.dtu.dk/services/NetMHCpan-3.0.

## Additional files

Additional file 1: Predictive performance on different alleles and peptide lengths for the allmer and 9mer predictive methods. The two methods were trained as described in the text. The predictive performance was measured in terms of Pearson’s correlation coefficient (PCC) and area under the ROC curve (AUC), the latter using a binding threshold of 500 nM. (XLSX 70 kb) Additional file 2: Predictive performance on different alleles and peptide lengths for the allmer and allmer-allele predictive methods. The two methods were trained as described in the text. The predictive performance was measured in terms of Pearson’s correlation coefficient (PCC) and area under the ROC curve (AUC), the latter using a binding threshold of 500 nM. (XLSX 66 kb) Additional file 3: Allele-specific length preference for 24 MHC molecules characterized by 20 or more ligand data points for the allmer and 9mer prediction methods compared to the length preference in the SYFPEITHI data. Length profiles for the allmer and 9mer methods were estimated as described in the text. (XLSX 50 kb)
